# Community’s Emergency Preparedness for Flood Hazards in Dire-dawa Town, Ethiopia: A Qualitative Study

**DOI:** 10.1371/currents.dis.3843ad9fc823c8c853970148b350750c

**Published:** 2018-02-21

**Authors:** Luche Tadesse Ejeta

**Affiliations:** Independent Public Health Consultant, Program Evaluation and Research, and Ethiopian Public Health Association, Addis Ababa, Ethiopia

## Abstract

**Background:**

Emergency preparedness at all levels (individuals and communities) is the corner stone of effective response to the increasing trends of global disasters due to man-made and natural hazards. It is determined by different factors, including (among others) past direct and indirect exposures to hazards. This study was carried out in Dire Dawa town, Ethiopia, which in the past experienced frequent flooding events, yet dearth of information exists about preparedness in the area.  The aim of the study was to assess the levels of emergency preparedness for flood hazards at households and communities levels.

**Methods:**

The study was conducted in a qualitative approach and was conducted in Dire Dawa town, which has been divided into nine administrative-units called Kebeles. Two focus group discussions were held in two of these units (Kebele-05 and 06), each focus group comprising twelve people (all above 18 years of age), and in total 24 people (13 females and 11 males) took part in the study. Open ended questions were used that could guide the discussions, and the discussions were audio-taped and transcribed. The results were translated from local language to English and qualitatively presented.

**Results:**

The findings of focus group discussions showed that the local government in collaboration with the federal government built the flood protection dams in areas where flood hazards have been thought to be repeatedly wreaking havoc, specifically after the flood disaster of the year 2006. In addition, in Kebele-05, where one Non-Governmental Organization (NGO) was operating on flood hazards prevention and mitigation program, some non-structural emergency preparedness measures were undertaken by the communities. These non-structural measures (the major ones) entailed: establishment of committees recruited from residents and training them to raise awareness among communities on emergency preparedness; some residents made changes to their own houses (retrofitted) and put sandbags around their houses to temporarily protect the flooding; establishment of communication channels between communities to alarm each other in the event of flood disaster; and reforestation of the already deforested mountainous areas surrounding the town. However, concerns were raised by study participants about strengths of the constructed flood protection dams. Furthermore, the non-structural emergency preparedness measures identified by this study were not comprehensive; for example, residents were not trained in first aid, first aid kits were not provided, there was no linkage being established between communities and health facilities so as to provide emergency medical care to victims in the event of flood disaster.

**Discussion:**

The findings of this study concur with some of the previous quantitative studies’ results in that the past direct and indirect disaster experiences invoke preparedness intention and actual preparedness for flood hazards at individuals, communities and organizations levels. The only one quantitative and behavioral based study conducted thus far in Dire Dawa town reported the strong association of past flood disaster experience with household emergency preparedness. Among the residents there was a tendency to rely on the dams to be constructed with “good quality” and “higher strength” than making preparedness efforts on their own at their households. Structural measures such as building of dams, dikes, levees, and channel improvements could be means of mitigation measures; however, solely relying on these measures could have far reaching consequences.

**Conclusions:**

To mitigate flood hazards, dams were built and in addition, in Kebele-05 where an NGO was operating, some non-structural emergency preparedness measures were undertaken. In the course of construction of flood protection dams, ensuring communities**’** involvement is needed**; **and at the same time undertaking comprehensive non-structural emergency preparedness measures in all Kebeles is highly recommended.

**Key words:**

Emergency, Preparedness, Flood, Dire Dawa, Ethiopia.

## INTRODUCTION

The global increasing trends of natural disasters makes preparedness for natural hazards a corner stone of effective response to disaster events (should they happen), and preparedness saves the loss of human lives and properties 1^,^^2^. Depending on the hazard type, preparedness (informed by scientific evidence) at community, individual and organization levels is recommended 3. Therefore, to help individuals and communities get prepared for hazards, provision of information or education is needed along with the necessary skills and resources. However, giving information to communities doesn’t necessarily lead to disaster preparedness 4 and studies have found that levels of preparedness at households and communities are low despite provision of information to communities and individuals and acknowledgement of risks by the vulnerable residents 5^,^^6^^,^^7^^,^^8^.

Hence, comprehending as to what motivates individuals to get prepared for natural hazards and what determines the preparedness behaviors (particularly those at risk of natural hazards) is quite a complex issue. Since preparedness has to do with human behaviors, a number of studies have been conducted applying behavioral theories to disaster and emergency health preparedness so as to better understand factors associated with preparedness behaviors 9. These theories mainly include the Health Belief Model (HBM), the Extended Parallel Process Model (EPPM), the Theory of Planned Behavior (TPB) and the Social Cognitive Theories 9. The constructs of these and other theories have been mainly made up of cognitive and affect ('positive or negative evaluations of object, behavior or idea’) factors at individuals level, and social factors at communities level, which all in turn are responsible for risk perception primarily emanating from past hazard exposure experiences 5^,^^10^^,^^11^^,^^12^. Despite the complexities, other studies too reaffirmed the importance of risk perception, prior experience, and better information about the protective mechanisms on preparedness behaviors 13^,^^14^. Therefore, prior direct or indirect experiences about hazards are very important for risk perception, which ultimately determines the disaster preparedness behavior status. Studies from developing countries, where shortage of information exists, on how the prior experience may influence the preparedness behaviors of individuals and communities could help to enrich the existing literature.

Ethiopia has been experiencing a number of natural disasters and associated consequences, chiefly due to drought and flood hazards. Flood hazards rank second (next to drought) in Ethiopia and they claimed a significant number of human lives and properties in the past, for which preparedness is highly needed. The frequency of flood disasters and their impacts are increasing over time, the most catastrophic flood disasters across the country being documented in the year 2006-killing an estimated of over 600 people and affecting more than 500,000 people 15. Injuries, disruption of pure water supply and sewage disposal systems (that potentially predispose the population to diarrheal diseases) and malaria epidemic outbreaks are the major consequences of flood disasters reported in Ethiopia 16.

Dire Dawa, a town with a current population of 293,000 (projected/estimated by Ethiopian Central Statistics Agency for the year 2017) 17 and located at 515 KM from the capital city to the east, is one of the areas prone to flood disasters in Ethiopia. The town has experienced flood disasters repeatedly, and the flood disaster of 2006 was the most devastating one in terms of claiming human lives and economic losses. It caused 256 deaths, resulted in 244 people missing, and displaced 10,000 people 18. Due to the fact that paucity of information exists in the country on preparedness for natural hazards in general and on flood hazards in particular, this study was conducted in the Dire Dawa town. Thus, the main aims of this study were to assess the levels emergency preparedness for flood hazards at the households/individuals and communities using exploratory method, and to assess how emergency preparedness for flood hazards has been influenced by the past disaster experiences and provision of information/education (if any) by external organizations (beyond the domain of local communities).

## METHODS

**Study setting and data collection**

The data for this study was collected on June 29 and 30, 2015, from Dire Dawa town (Ethiopia), which is located in eastern part of the country. The town has nine administrative units called Kebeles and each Kebele has its own distinct population and administrative office. Two focus group discussions were held in two of the Kebeles (Kebele 05 and 06), whereby in the past they frequently experienced flood disasters and have been more prone to flood hazards. Each focus group discussion comprised of twelve people (all above 18 years of age). From Kebele-05, six males and six females participated, while from Kebele-06, five males and seven females took part in the study. Participants were recruited and included into this qualitative study mainly based on the knowledge of socio-demographic, familiarity with the study area, knowledge of tradition and culture of the town, and regularity of participating in community based activities including disaster risk reduction initiatives. Study participants were not categorized according to their ethnic groups and religious denominations as selection criteria, however, the participants were from the dominant ethnic groups and religious denominations of both Kebeles.

Open ended questions were prepared that could guide the two focus group discussions. These questions were about whether the town was vulnerable to flood hazards, experiences of flood related losses in the past, whether the risk of flood hazards could be averted through preparedness actions at households and communities levels, if there were any prevention and mitigation measures undertaken by government or non-governmental organizations, and if the residents were feeling safe as a result of emergency preparedness (prevention and mitigation) actions undertaken at households and communities levels (please refer to annex for the full open ended questions). The focus group discussions were audio-taped and transcribed; the results were translated from local language to English and qualitatively presented.

**Ethical consideration**

Ethical approval was obtained from the Ethiopian Ministry of Science and Technology, which is the responsible body in the country for ensuring the ethical standards of studies, and permission to conduct the study was obtained from Dire Dawa Administrative Council Health Bureau and from each Kebele Administrative Office. Information was provided to participants about the purpose of the study and participants were informed that taking part in the study was solely based on their willingness. The consent sought was verbal, because some of the study participants couldn't read and write. The ethics committee from Ethiopian Ministry of Science and Technology approved this verbal consent procedure. The study participants verbally consented to their opinions including direct quotations published without revealing their personal identities. After all study participants agreed to take part in the study and verbally consented for the discussions to be audio-taped, the focus group discussions were held and audio-taped. The verbal consent procedure was documented by the author.

## RESULTS

Given the repeated flood disasters that happened in Dire Dawa town in the past, all the participants of the focus group discussions agreed that Dire Dawa town was vulnerable to flood hazards, though some of them argued that its exposure level to flood hazards lessened in the recent times owing to the construction of flood protection dams. Furthermore, the participants stated that major flood disasters events were witnessed in the year 1981, 1997, and 2006. From these events, the year of 2006 flood disaster was reported to be the most devastating one in the history of Dire Dawa town-in terms of claiming human lives and loss of properties. A number of reasons were cited by participants that could increase the vulnerability of the town to flood hazards, and the main ones were: topography of the town (relative to its surrounding districts, Dire Dawa town is situated in lower altitude and surrounded by mountains), deforestation in mountainous areas surrounding the town, and construction of houses in the vicinity of ditches and rivers (that cross the city during rainy season), contributing to the narrowing of the flood-ditches and rivers’ ways, often causing flooding due to the overflow of water into the nearby households. One of the participants from Kebele-05 put the vulnerability of Dire Dawa town to flooding in this way: *“it is possible to categorize into three groups about the vulnerability of Dire Dawa town: firstly, because of deforestation, there is nothing that guards against the rivers that flow from the upper stream to the town and they [rivers] simply overflow into the town; secondly, previously the mountains were filled with forests, now all those forests have been cleared and when it rains, it directly comes down to the town; and third, there are houses built on the ways of floods (ditches) that caused the narrowing of the ditches, and floods that supposed to go through those ditches overflow into the nearby houses”.*

According to the views of the participants, after the major flood disaster event of 2006, some actions had been taken to reduce the vulnerability of the town to flood hazards. These actions were: construction of flood protection dams (in both Kebeles), organizing communities for planting various trees in the mountainous areas surrounding the town and in previously deforested places (Kebele-05), increasing the awareness level of communities on flood protection measures and on communications between communities of different villages (Kebele-05). In addition, by virtue of experiencing past flood disasters, the residents of the town had made some changes to their own respective houses and the surroundings (Kebele-05). These changes entailed mainly putting sandbags around their houses to protect the flooding, and construction around their homes to elevate the floors higher above the normal surface in which the floods run, which were ultimately meant to make the houses a little bit “flood-resistant”. However, until the question was posed to them, whether they had done something to their homes and the surroundings on their own initiative or not, they were repeatedly raising about construction of dams to protect and mitigate the flood disasters, which shows the residents were heavily putting their hopes on constructed or yet to be constructed flood protection dams. This pattern was similar in both group discussions (Kebele-05 and 06).

The flood hazards prevention and mitigation actions were carried out by the initiatives of the government, a non-governmental organization operating in the town and by residents of the town. The government built dams after the major flood disaster of 2006, and the participants said that construction of dams had somewhat reduced risks associated with flood disasters. For example a participant of focus group discussion from Kebele-06 being heard of saying, *“since dams are being constructed in challenging and difficult places where floods are thought be causing problems, I don’t think the flooding in the town would create the likes of problems it caused in the past”.* And the other participant from the same Kebele added,* “it is clear to everyone that the past disasters left behind terrible memories, after those periods, both government and the community have been making efforts [to prevent and mitigate flood hazards]; however, it is impossible to say ‘reliable’.’’* Therefore, participants of focus group discussions from both Kebeles repeatedly raised their concerns surrounding the strengths of these dams as some of these dams had already been demolished by relatively what participants called, “smaller floods”.

According to the focus group discussion held in Kebele-05, there was an indigenous non-governmental organization called “Jerusalem Children and Community Development Organization (JeCCDO)”, at the time of the data collection this organization was registered as “an Ethiopian Residents’ Charity Organization”, rendering services to communities on flood disaster prevention and mitigation activities. On accounts of participants, the services that JeCCDO was offering include: organizing the communities and establishing committees recruited from four villages of Kebele 02 and 05, building the capacity of these committees through training that could enable them to carry out awareness raising activities on flood disaster prevention and mitigation. As a result, trees were planted in areas previously deforested, communication channels were created between communities of the upper and lower streams so that residents of the upper stream could inform the residents in the lower stream by mobile phones when flooding was about to come. However, concerns were raised again on the means of communication, for instance, one woman from Kebele-05 said, *“this means of communication is not that much satisfactory and sustainable, because during flooding, during rainy times, there are times when the telephone/the mobile itself not functioning”*. In kebele-05’s group discussion, study participants also raised the presence of ‘bell’ in the Kebele-05 Administrative Office to ring it in the event of flooding as a warning for evacuation, and the presence of gun firing into the air by police, this too, aimed to alarm the residents. On the other hand, in Kebele-06, participants said that most of the residents in that area were too poor to make changes to their homes on their own and community based initiatives of flood hazards prevention and mitigation efforts were not mentioned. In addition, participants said that apart from the government there was no other non-governmental organization operating in Kebele-06 on flood hazards prevention and mitigation program.

Participants were asked to what extent they and their families were relying on the preventive and mitigation measures being undertaken at households and communities levels thus far (at the time of data collection). And their responses were that they couldn’t rely on those measures-*“we cannot say that we are threat free from the flood disaster, because every time situations are changing”*, said one man from Kebele-05; the other person from the same Kebele added, *“dams have been constructed, but it is impossible to rely on these dams, because just recently last time it was only the ‘smaller floods’ that managed to demolish and take away those dams”*; a women from the same Kebele further added,* “the dams were constructed long before the establishment of committees (the committees were established by JeCCDO in order to work on prevention and mitigation activities in Kebele 02 and 05) and they (dams) do not have quality, and it would have been good if committees were consulted and involved in the construction process”*; and another man from Kebele-05 voiced his worries in saying,*” we have repeatedly expressed our concerns to the Kebele administration and Dire Dawa town Administrative Council about the threat of flood disasters”*. In general, participants from both focus groups stressed that the constructed flood protection dams had no quality and strengths, and these dams were not something to be reckoned with. And they recommended that the residents of the Dire Dawa town should be involved and allowed to be part of the construction process of the dams so as to ensure the strengths of these dams and to avoid the catastrophic situation that may come in the near future due to flood disasters. Moreover, like any other cities (towns) in Ethiopia, there are people residing on streets, and in the past, streets dwellers were also the victims of flood disasters in Dire Dawa town, and participants (particularly from Kebele-06) recommended that shelters should be provided to those people. In addition, the group discussion’s participants of Kebele-06 acknowledged that there were no communications between the communities and they pointed out the necessity for the establishment of committees in their Kebele that would convene meetings on a regular basis to discuss and share information among the residents on how to prevent and mitigate the food hazards.

## DISCUSSION

This study used a qualitative approach (focus group discussions) to investigate the levels of preparedness at individuals and communities in the town at risk of flood hazards. Furthermore, it was aimed at assessing if the previous flood disasters might have influenced the residents, the local and federal governments, and other organizations in taking some proactive emergency preparedness actions in preventing and mitigating future flood disasters in the area. The findings of focus group discussions show that the local government (Dire Dawa Administrative Council) in collaboration with the federal government built the flood protection dams in areas where flood hazards have been thought to be repeatedly wreaking havoc, specifically after the flood disaster of the year 2006. Even though the findings were controversial among studies, the findings of this study concur with some of the previous quantitative studies’ results in that the past direct and indirect disaster experiences invoke preparedness intention and actual preparedness for flood hazards at individuals, communities and organizations levels 5^,^^19^^,^^20^^,^^21^. The only one quantitative and behavioral based study conducted thus far in Dire Dawa town reported the strong association of past flood disaster experience with household emergency preparedness 21.

However, according to the views and observations of study participants, the strengths of the built flood-protection dams have been under question whether they could really protect the residents from heavy flood hazards or not as some of the dams had already collapsed due to relatively “smaller floods”. Hence, the results of the focus group discussions from Kebele-05 and 06 demonstrate that the residents of the Dire Dawa town did not have confidence in the constructed dams and the participants from both groups raised their concerns about the strengths of already built dams, and calling and recommending for the dams to be constructed with “high quality and strength”. However, the participants didn’t raise any other alternative means of saving their own lives and properties until the question was posed to them whether they had done anything at individuals’ level on their own or by the initiative of other external organizations. This means the residents were putting much of their hopes primarily on the dams to be constructed with “good quality” and “higher strength” than making preparedness efforts on their own at their households. Structural measures such as building of dams, dikes, levees, channel improvements could be means of mitigation measures; however, solely relying on these measures could have far reaching consequences if alternative preparedness of non-structural emergency measures are not put in place as these structural mitigation measures may fail when the hydrological load exceeds their capacities 22^,^^23^^,^^24^^,^^25^. And hence, from the view point of cost implication to put in place the structural measures to mitigate flood hazards might not be affordable always and due to the fact that there might be unforeseen consequences of relying on these measures (even if afforded), emergency preparedness involving non-structural measures will pay off in the long run.

Nevertheless in kebele-05, where there was a community based non-governmental organization working on flood hazards prevention and mitigation program at the time of data collection, there were communications between the communities through the established committees about prevention and mitigation, preparedness at the households and communities levels existed, and the communities were better informed about flood hazards prevention and warning systems. As this study employed the qualitative approach, study participants were not requested to rate their responses of preparedness measures they undertook as often being done by relatively quantitative studies. Even though the study participants of kebele-05 didn’t characterize the level of non-structural preparedness measures in terms of scale or number, a local NGO operating in the Kebele-05 has stated the extent of some of these measures in its annual report of the year 2015. According to this report, for example, “37 community leaders of disaster prone areas networked to run Community Based Early Warning system”, 2822 people (1,095 females) received “awareness raising information” on disaster risk reduction through different forms of activities, “2 community owned and managed nursery sites [were] established and strengthened to support afforestation programs”, and “20 hectares of land rehabilitated through soil and water conservation practices” 26. As mentioned in the result part, this local NGO’s name was “Jerusalem Children and Community Development Organization (JeCCDO)”; at the time of the data collection this organization was registered as “an Ethiopian Residents’ Charity Organization” providing services to communities on disaster risk reduction and others 26. On the contrary, in Kebele-06, such community based preparedness initiatives were not mentioned by participants and it looks they are lacking. This shows even though the communities experienced the flood disasters in past repeatedly, provision of information is needed on better emergency preparedness, particularly not only about the risk of flood hazards but also about the ramifications of flood disasters and the cost effective methods of mitigation measures at households levels 25.

In general, this exploratory study found out that apart from the preparedness efforts put in place by the government, non-governmental organization, and the communities that were identified by these focus group discussions, there were no other essential emergency preparedness activities. For example, there were no linkages established between the health facilities and the communities to provide emergency medical care in the event of flood disaster, training were not provided to communities’ members on first aid and first aid kits were not given to them, there were no routes of evacuation plans and temporary shelters. Hence, more work remains to be done to engage communities in structural and non-structural flood hazards mitigation measures (early warning, emergency and precautionary measures among others).

**Limitations of the approach**

The most commonly recommended number of participants for a single focus group discussion is from 8 to 12 people, and in this study 12 individuals took part in each discussion, and even some qualitative research text books put the maximum figure as 8 individuals 27. Hence, the sample size of this study per focus group discussion was adequate. From nine existing kebeles in the town, two kebeles were selected purposely based on their exposure status to flood hazards, which could be one of the limitations of this study. Another limitation of this study could be only focus group discussions were employed instead of mixing up with other qualitative study types such as “an individual based in-depth interview”, which could have enriched the information on disaster preparedness status. Future qualitative studies to be conducted in the town could benefit if they address these limitations.

## CONCLUSIONS

In the history of Dire Dawa town, the flood disaster of 2006 was the turning point in prompting some emergency preparedness actions. The local government in collaboration with the federal government initiated the construction of flood protection dams in some flood prone areas of the town. However, according to this study’s findings, some of the already constructed dams had already collapsed and the residents had no confidence in those dams. In Kebele-05 where an NGO existed to operate on flood hazards prevention and mitigation, some emergency preparedness actions were undertaken by the communities. These major non-structural measures undertaken include: putting sandbags around households for temporary mitigation, making changes around homes, planting of trees in the mountainous areas surrounding the town, establishment of communication channels between different communities to notify each other during the event of flood disaster, and the existence of flood disaster “means of warning” at the Kebele level. Involvement of communities in the course of construction of flood protection dams to ensure dams’ strengths, and provision of comprehensive information on non-structural measures of emergency preparedness (including linking the emergency preparedness with the health facilities for emergency medical care during disaster event and having routes of evacuation plans) are recommended, particularly in Kebeles other than Kebele-05, where non-governmental organizations are inactive on flood prevention and mitigation program.

## DATA AVAILABILITY

The original audio-taped interviews were transcribed and translated from the local language to English, it can be accessed at: 10.6084/m9.figshare.5853582, https://figshare.com/articles/Transcriptions_pdf/5853582.

## COMPETING INTERESTS

The author has declared that no conflicts of interest exist.

## CORRESPONDENCE

Luche Tadesse Ejeta, BS, MPH, PhD, e-mail: tadesse.ejeta@gmail.com

## SUPPORTING INFORMATION

**Appendix I:** Questions for Focus Group Discussions

No	Question
1	In your opinion, do you think that Dire Dawa town is prone to flood disaster? If yes, why?
2	When did the major flood disaster happen in Dire Dawa town? Why you think that was the major flood disaster in the history of Dire Dawa town?
3	Do you think that the residents of Dire Dawa town are at risk of flood disaster that might happen during rainy season in the near future? If you think that the residents are at risk, why?
4	Do you think the flood disaster could be prevented or mitigated through some actions by human being in your area?
5	To avert the flood disaster risk, are there any specific things you have done around the home to minimize the effects of flooding? (Why did you/why didn’t you?)Where did you get this information from?Why did you think this information was useful?
6	Have you discussed flood issues with others? (Why did you/why didn’t you?)What/who influenced you to discuss these issues?Did this help in clarifying issues?Would it influence you to become more involved in the wider community?
7	Have you had any contact with the Dire Dawa council or the local office (kebele) and any health facilities (hospitals or health centers) regarding floods?Have they contacted you?How satisfactory was this contact?
8	Is there any action taken by the government (could be local or federal) to mitigate the flood disaster in your area? If yes, what are those actions?
9	Is there any action taken by the non-governmental organizations (could be civil society organizations, local-NGOs, and International NGOs) to mitigate the flood disaster in your area? If yes, what are those actions?
10	How much do you rely on the preventive and mitigation measures being undertaken by you personally at household level, government, and non-governmental organizations? Do you feel that you and your families and the community at large are safe as result of these actions (individually, government and non-governmental organizations)?
